# Single-nucleotide polymorphisms as important risk factors of diabetes among Middle East population

**DOI:** 10.1186/s40246-022-00383-2

**Published:** 2022-04-02

**Authors:** Iman Akhlaghipour, Amir Reza Bina, Mohammad Reza Mogharrabi, Ali Fanoodi, Amir Reza Ebrahimian, Soroush Khojasteh Kaffash, Atefeh Babazadeh Baghan, Mohammad Erfan Khorashadizadeh, Negin Taghehchian, Meysam Moghbeli

**Affiliations:** 1 Student Research Committee, Faculty of Medicine, Mashhad University of Medical Sciences, Mashhad, Iran; 2 Student Research Committee, School of Medicine, Birjand University of Medical Sciences, Birjand, Iran; 3 Student Research Committee, Faculty of Dentistry, North Khorasan University of Medical Sciences, Bojnurd, Iran; 4 Medical Genetics Research Center, Mashhad University of Medical Sciences, Mashhad, Iran; 5 Department of Medical Genetics and Molecular Medicine, School of Medicine, Mashhad University of Medical Sciences, Mashhad, Iran

**Keywords:** Diabetes, Single-nucleotide polymorphism, Diagnosis, Middle East

## Abstract

Diabetes is a chronic metabolic disorder that leads to the dysfunction of various tissues and organs, including eyes, kidneys, and cardiovascular system. According to the World Health Organization, diabetes prevalence is 8.8% globally among whom about 90% of cases are type 2 diabetes. There are not any significant clinical manifestations in the primary stages of diabetes. Therefore, screening can be an efficient way to reduce the diabetic complications. Over the recent decades, the prevalence of diabetes has increased alarmingly among the Middle East population, which has imposed exorbitant costs on the health care system in this region. Given that the genetic changes are among the important risk factors associated with predisposing people to diabetes, we examined the role of single-nucleotide polymorphisms (SNPs) in the pathogenesis of diabetes among Middle East population. In the present review, we assessed the molecular pathology of diabetes in the Middle East population that paves the way for introducing an efficient SNP-based diagnostic panel for diabetes screening among the Middle East population. Since, the Middle East has a population of 370 million people; the current review can be a reliable model for the introduction of SNP-based diagnostic panels in other populations and countries around the world.

## Background

**Fig. 1:**
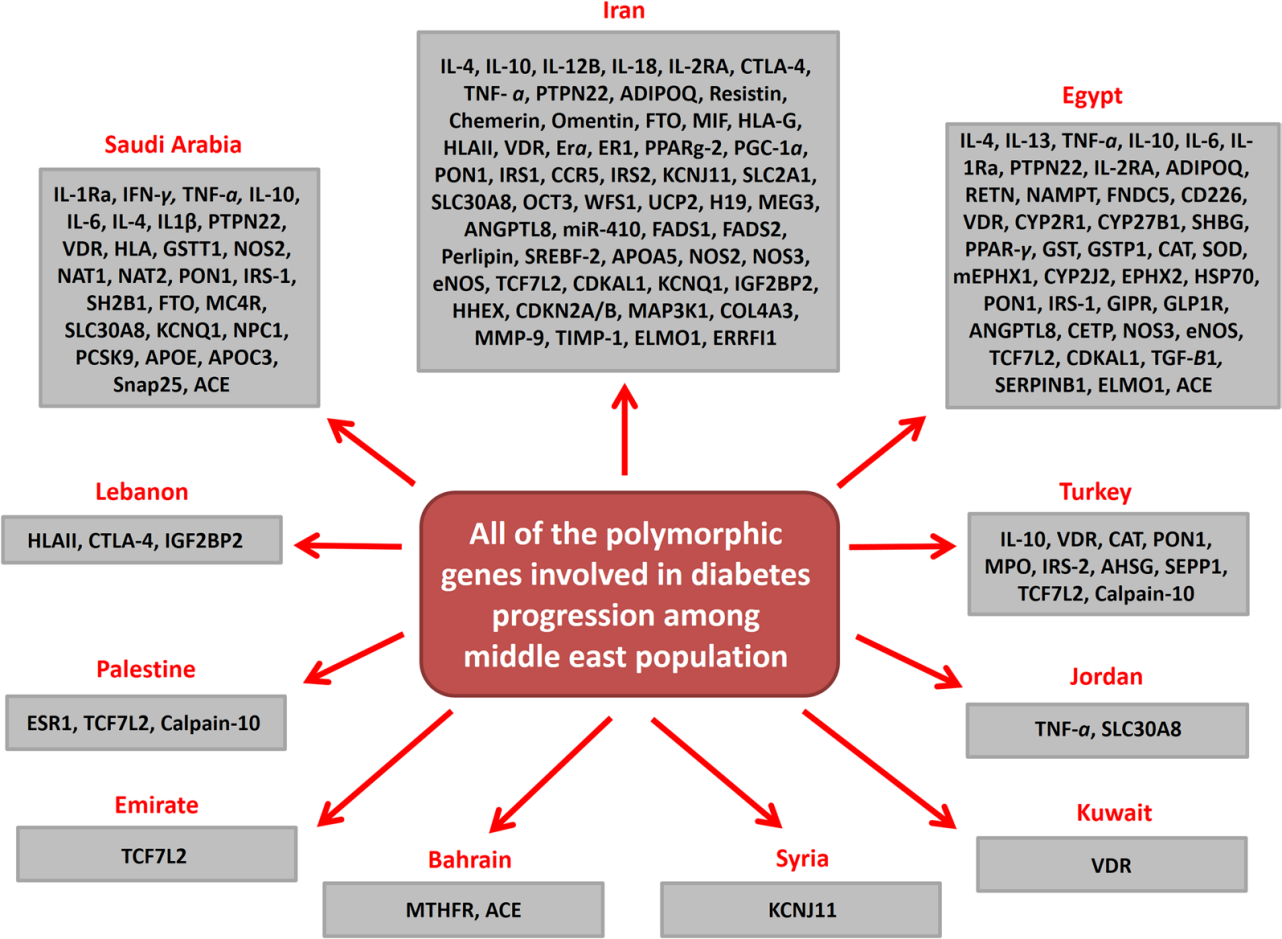
All of the polymorphic genes that have been involved in diabetes progression among Middle East population

Endocrine disorders are the fifth leading cause of death in the world [1]. Diabetes mellitus is known as the most common endocrine disorder that occurs through hyperglycemia following the deficiency of insulin production or function [2]. It can be classified into three main types including; gestational diabetes mellitus (GDM), type 1 diabetes (T1D), and type 2 diabetes mellitus (T2D) [3]. T1DM and T2DM are proved to be the most prevalent types of diabetes [4–6]. GDM is one of the most important metabolic disorders during pregnancy that is observed in about 7% of all pregnancies [7]. The persistent hyperglycemia affects the normal function of multiple organs such as eyes, kidneys, and cardiovascular system [3]. Due to its high prevalence, diabetes mellitus is regarded as a global health challenge [8]. During the past three decades, the prevalence of diabetes has increased notably in low- and middle-income countries. The Eastern Mediterranean is one of the hot spots of diabetes with about 13.7% of affected adults [9]. Middle East and North Africa had the highest prevalence of diabetes (12.2%) in 2019 that is expected to have a 96% increase until 2045. However, in the same period of time it is expected to observe increased diabetes prevalence by 15% in Europe [10]. Kuwait and Yemen had the highest and lowest diabetes prevalence with 15.4% and 6.8%, respectively, in Middle East in 2000. All of the Middle East countries experienced elevated prevalence of diabetes between 2000 and 2014. Besides the genetic predisposition, various other factors such as obesity-related physical inactivity, poor nutritional habits, and urbanization are also involved in the rising trend of diabetes prevalence in the Middle East. Kuwait, Qatar, and Egypt were the top ranks of diabetes prevalence among Middle East countries between 2000 and 2014 [11]. It has been observed that the prevalence of diabetes was 11.4% among Iranian population with an annual incidence of 1% [12]. It is expected that approximately 9.2 million Iranians suffer from diabetes by 2030 [12]. Diabetes is a heterogeneous disorder affected by a wide range of genetic and environmental factors. Despite the population heterogeneity in Middle East regarding the ethnic, income, and socioeconomic status, various risk factors are involved in diabetes such as aging, lifestyle change, reduced physical activity, and high calorie diet [13]. Genetic factors can also be associated with increased diabetes susceptibility [14, 15]. Various genes are involved in the molecular mechanism of diabetes progression. A single-nucleotide polymorphism (SNP) or single-gene mutation has not the same results between different individuals and populations. This difference is directly or indirectly influenced by the overall genetic background related to the individual, family, or population that are potentially interacted with variety of environmental factors [16]. Genome-wide association studies identified 70 loci in different populations related to T2D and demonstrated an association between SNPs and T2D susceptibility [17]. Majority of the diabetic patients have not any significant clinical manifestations in the primary stages of diabetes that results in severe tissue damages in various organs such as kidney and eyes. Regarding the importance of genetic changes as pivotal risk factors associated with diabetes susceptibility, we examined the role of SNPs in the pathogenesis of diabetes in the Middle East population (Fig. 1; Table 1). The aim of present review is to assess the molecular pathology of diabetes in the Middle East population that paves the way for introducing an efficient SNP-based diagnostic panel for diabetes screening among the Middle East population.

**Table 1:** All of the SNPs associated with diabetes susceptibility among Middle East population

Study	Year	Population	Gene	SNP	Sample size	Odds ratio (OR)
Ali [24]	2015	Saudi Arabia	IL-1Ra	VNTR	100 T1D children 102 healthy controls	OR = 1.97
Alsaid [26]	2013	Egypt	IL-4 and IL-13	− 590 C>T and -1112 C>T	135 T2D patients 75 healthy controls	OR = 6.27 OR = 4.57
Ali [27]	2018	Egypt	IL-4	VNTR	102 T2D patients 188 healthy controls	
Kazemi Arababadi [31]	2010	Iran	IL-4	− 590	100 T2D patients 150 healthy controls	
Arababadi [33]	2012	Iran	IL-10	− 592	100 T2D patients without nephropathy 100 T2D patients with nephropathy 100 healthy controls	
Erdogan [34]	2012	Turkey	IL-10	− 1082G/A	91 T2D patients 112 healthy controls	
Yaghini [35]	2012	Iran	IL-12B	+ 1188	114 T2D patients 100 healthy controls	OR = 0.3
Mojtahedi [38]	2006	Iran	IL-18	− 607 A/C and − 137 C/G	112 T1D patients 194 healthy controls	
Ranjouri [40]	2016	Iran	IL-2RA and CTLA4	ss52580101C>A and + 49A/G	50 T1D patients 50 healthy controls	
Mojtahedi [41]	2005	Iran	CTLA-4	+ 49 A/G	109 T1D patients 331 healthy controls	
Kiani [42]	2016	Iran	CTLA-4	− 1722 (T/C), − 318 (C/T), and + 49 (G/A)	111 T2D patients 100 healthy controls	
Ei Wafai [43]	2011	Lebanon	HLAII and CTLA-4	HLA (DQB1 and DRB1) and CTLA-4 (A49G)	39 T1D patients 46 healthy controls	OR = 3.381
Settin [47]	2009	Egypt	TNF-α, IL-10, IL-6, and IL-1Ra	− 308 G/A, − 1082 G/A, − 174 G/C and VNTR	50 T1D patients 98 healthy controls	OR = 7.91 OR = 3.36 OR = 3.68
Golshani [48]	2015	Iran	TNF-α	− 308 G/A	1038 T2D patients 1023 healthy controls	OR = 2.34
Allam [49]	2018	Saudi Arabia	IFN-γ, TNF-α, IL-10, IL-6, IL-4, and IL-1β	rs2430561, rs1800629, rs1800872, rs1800796, rs2243250, and rs16944	300 T1D patients 300 healthy controls	OR = 1.28 OR = 1.73 OR = 2.23 OR = 2.24 OR = 1.85
Emara [50]	2020	Egypt	TNF-α	− 1031T/C	78 T2D patients 20 healthy controls	OR = 2.446
Al-Azzam [51]	2014	Jordan	TNF-α	G-308A	355 T2D patients	
Alswat [54]	2018	Saudi Arabia	PTPN22	C1858T	372 T1D patients 372 healthy controls	OR = 4.4
Abbasi [55]	2017	Iran	PTPN22	rs12760457, rs1310182, rs1217414, rs33996649, and rs2476601	99 T1D patients 100 healthy controls	
Abdelrahman [57]	2016	Egypt	PTPN22 and IL-2RA	rs2476601 and rs11594656	150 T1D patients 165 healthy controls	OR = 2.2
Nomani [62]	2019	Iran	ADIPOQ	− 11,377 C/G and − 11,391 G/A	189 (100 T2D and 89 T1D) patients 161 healthy controls	
Mohammadzadeh [63]	2009	Iran	ADIPOQ	+ 45 T/G and + 276 G/T	50 T2D patients 52 healthy controls	OR = 2.574
Takhshid [64]	2015	Iran	ADIPOQ	rs2241766	65 GDM patients 70 healthy controls	OR = 2.38
El-Shal [65]	2014	Egypt	ADIPOQ	+ 45 TG, GG, − 11,391 AA, and + 276 TT	296 T2D patients 209 healthy controls	OR = 1.92 OR = 3.52 OR = 1.83 OR = 4.2 OR = 0.48
Takhshid [67]	2015	Iran	Resistin	− 420C>G	75 GDM patients 70 healthy controls	
El-Shal [68]	2013	Egypt	RETN	+ 299 AA and − 420 GG	145 patients 155 healthy controls	
Motawi [69]	2014	Egypt	NAMPT and RETN	− 948G/T and − 420C/G	90 T2D patients 60 healthy controls	
Hasanvand [72]	2018	Iran	Chemerin	rs17173608 and rs4721	130 GDM patients 160 healthy controls	OR = 2.3 OR = 2.21
Khoshi [74]	2019	Iran	Omentin and FTO	rs2274907 and rs9939609	83 T2D patients 85 healthy controls	OR = 1.98 OR = 2.57
Khidr [77]	2017	Egypt	FNDC5	rs16835198 G>T	100 T2D patients 50 healthy controls	
Hamidi [79]	2019	Iran	MIF	− 173 G>C (rs755622)	120 T2D patients with depression 120 T2D patients without depression	
Abu El-Ella [82]	2018	Egypt	CD226	rs763361 C>T	74 T1D patients 82 healthy controls	OR = 1.68
Rezaei [89]	2021	Iran	HLA-G	HLA-G 14-bp Insertion/Deletion	102 pancreas transplant recipients 100 normal controls	OR = 3.82
Mansoori Derakhshan [92]	2015	Iran	HLAII	DRB1*0301, DQA1*0501 and DQB1*0201	80 T1D patients 80 healthy controls	OR = 1.81 OR = 4.68 OR = 2.40
Mohammadnejad [110]	2012	Iran	VDR	FokI, BsmI, ApaI, and TaqI	87 T1D patients 100 healthy controls	OR = 0.51
Rahmannezhad [111]	2016	Iran	VDR	rs7975232 and rs731236	157 GDM patients 157 healthy controls	OR = 2.996
Razi [112]	2019	Iran	VDR	rs7975232, rs731236, and rs4516035	104 diabetic patients with nephropathy 100 diabetic patients without nephropathy 109 healthy controls	
Aslani [113]	2011	Iran	VDR	FokI	142 GDM patients 161 healthy controls	
Abd-Allah [114]	2014	Egypt	VDR	BsmI, FokI, ApaI, and TaqI	120 T1D patients 120 healthy controls	OR = 2.3 OR = 2.2 OR = 1.8 OR = 4.03
Ahmed [115]	2019	Egypt	VDR	rs7975232, rs731236 and rs1544410	50 T1D patients 50 healthy controls	OR = 2.8 OR = 4.38
Al-Daghri [117]	2012	Saudi Arabia	VDR and HLA	rs731236-AG, rs1544410-CT, and DRB1*04	368 T2D patients 259 healthy controls	
Al-Daghri [118]	2014	Saudi Arabia	VDR	Intron 8 (BsmI, ApaI) exon 9 (TaqI) and exon 2 (FokI)	285 Metabolic syndrome patients 285 healthy controls	OR = 1.7 OR = 1.5 OR = 0.70 OR = 0.67 OR = 0.73
Ali [119]	2018	Saudi Arabia	VDR	FokI and BsmI	100 T1D patients 102 healthy controls	OR = 1.9 OR = 2.5
Apaydın [120]	2019	Turkey	VDR	rs15444410, rs7975232, rs19735810 and rs731236	100 GDM patients 135 healthy pregnant controls	
Rasoul [121]	2019	Kuwait	VDR	rs10735810, rs731236, rs7975232, and rs1544410	253 T1D patients 214 healthy controls	
Hussein [124]	2012	Egypt	CYP2R1 and CYP27B1	rs10741657 and rs10877012	120 T1D patients 120 healthy controls	OR = 2.6 OR = 3.7 OR = 2.9
Mohammadi [129]	2013	Iran	ERα	PvuII and XbaI	174 T2D patients 174 healthy controls	OR = 0.67 OR = 0.061
Meshkani [130]	2012	Iran	ER1	PvuII and XbaI	155 T2D patients 377 healthy controls	OR = 0.22
Ereqat [132]	2019	Palestine	ESR1	PvuII and XbaI	102 T2D patients 112 healthy controls	OR = 2.03
El Tarhouny [134]	2015	Egypt	SHBG	rs6257 and rs6259	185 T2D patients 185 healthy controls	OR = 2.241
Meshkani [140]	2007	Iran	PPARg-2	Pro12Ala	284 T2D patients 412 healthy controls	OR = 0.395
Shokouhi [142]	2015	Iran	PGC-1α	Gly482Ser, Thr394Thr, and Thr528Thr	173 T2D patients 173 healthy controls	OR = 5.23 OR = 2.37
Hasan [145]	2017	Egypt	PPAR-γ	rs1801282	205 T2D patients 100 healthy controls	OR = 3
Barseem [155]	2017	Egypt	GST	T1/M1	64 T1D patients 41 healthy controls	OR = 4.2
Amer [157]	2012	Egypt	GSTP1	Ile105Val	112 T2D patients 188 healthy controls	
Gusti [158]	2021	Saudi Arabia	GSTT1 and NOS2	rs17856199 and rs2297518	177 T2D patients 207 healthy controls	OR = 3.42 OR = 3.57 OR = 4.06
Ghattas [167]	2012	Egypt	CAT and SOD	1167C/T and + 35 A/C	105 T2D patients 115 healthy controls	OR = 2.65 OR = 5.68 OR = 3 OR = 3.25 OR = 3.44
Ghattas [168]	2012	Egypt	mEPHX1	rs2234922 and rs1051740	112 T2D patients 150 healthy controls	
Habieb [169]	2020	Egypt	CYP2J2 and EPHX2	rs2280275 and rs751141	140 T2D patients 60 healthy controls	OR = 0.375 OR = 0.440 OR = 0.195 OR = 0.195
Elshahed [171]	2020	Egypt	HSP70	− 110 AC, + 190 G/C, + 1267 A/G, and + 2437T/C	60 T2D patients 30 healthy controls	
Al-Shaqha [175]	2015	Saudi Arabia	NAT1 and NAT2	rs1041983, rs1799931, rs1799930, rs1799929, and rs4986988	186 T2D patients 183 healthy controls	
Shakeri [179]	2017	Iran	PON1	− 108C>T	90 T2D patients 90 healthy controls	
Emami [180]	2018	Iran	PON1	− 108, − 126, and − 162	98 T2D patients 104 healthy controls	
Khajeniazi [181]	2020	Iran	PON1	− 108C>T	90 T2D patients 90 healthy controls	
El-Lebedy [182]	2014	Egypt	PON1	Q192R and L55M	68 patients with T2D 66 patients with T2D + CVD 50 healthy controls	
Al-Hakeem [183]	2014	Saudi Arabia	PON1	rs662	200 GDM patients 300 healthy controls	
Arpaci [184]	2020	Turkey	CAT and PON1	− 262 C/T and 55 L/M	100 T2D patients 100 healthy controls	
Ergen [188]	2014	Turkey	MPO	− 463 G/A	145 T2D patients 151 healthy controls	
Keshavarzi [190]	2019	Iran	IRS1 and CCR5	rs10498210 G/A and 59029 A/G	120 T2D patients 70 healthy controls	OR = 2.9 OR = 3.3
Golsheh [191]	2019	Iran	CCR5 and IRS1	59029A/G and rs10498210	220 T2D patients 200 healthy controls	OR = 1.9 OR = 2.62
Haghani [192]	2012	Iran	IRS-1 and IRS-2	Gly972Arg and Gly1057Asp	336 T2D patients 341 healthy controls	OR = 1.76 OR = 3.86 OR = 1.63 OR = 1.63 OR = 1.69 OR = 3.1 OR = 1.86 OR = 1.76 OR = 1.83 OR = 2.35
Yousef [193]	2018	Egypt	IRS-1	r.2963G>A (rs1801278)	100 T2D patients 120 healthy controls	
Ayaz [194]	2014	Turkey	IRS-2	G1057D	44 GDM patients 50 healthy controls	
Alharbi [195]	2014	Saudi Arabia	IRS-1	rs1801278	200 GDM patients 300 healthy controls	
Nemr [197]	2012	Lebanon	IGF2BP2	rs4402960 and rs1470579	544 T2D patients 606 healthy controls	OR = 1.43 OR = 5.49
Al-Hakeem [200]	2014	Saudi Arabia	SH2B1	rs4788102	200 GDM patients 300 healthy controls	
Rastegari [202]	2015	Iran	KCNJ11	E23K (rs5219)	20 diabetic patients 20 healthy controls	
Makhzoom [203]	2019	Syria	KCNJ11	rs5219	75 T2D patients 63 healthy controls	OR = 3.81
Akbas [209]	2020	Turkey	AHSG	− 843A>T (rs2248690) and 767C>G (rs4918)	83 GDM patients 100 healthy pregnant controls	
Akbaba [211]	2018	Turkey	SEPP1	rs4987017, rs13154178, rs146125471, rs28919926, and rs16872762	40 GDM patients 40 healthy pregnant controls	
Amini [213]	2016	Iran	SLC2A1	HaeIII	126 T2D patients with nephropathy 254 T2D patients without nephropathy	OR = 6.3905
Soltanian [214]	2020	Iran	SLC30A8	rs13266634	125 T2D patients 125 healthy controls	OR = 1.43
Mashal [217]	2010	Jordan	SLC30A8	rs13266634	358 T2D patients 326 healthy controls	OR = 1.47 OR = 2.44 OR = 1.64
Bazzi [218]	2014	Saudi Arabia	FTO, MC4R, SLC30A8, and KCNQ1	rs9939609 (A/T), rs17782313 (C/T), rs12970134 (A/G), and rs13266634 (C/T)	90 T2D patients 95 healthy controls	
Mahrooz [220]	2017	Iran	OCT3	rs3088442G>A and rs2292334G>A	150 T2D patients 150 healthy controls	OR = 0.016 OR = 0.61
Torkamandi [229]	2017	Iran	WFS1	rs1801214 and rs1046320	220 T2D patients 211 healthy controls	OR = 0.68
Rezapour [232]	2021	Iran	UCP2	45-bp ins/del	80 T2D patients 77 healthy controls	
Al-Daghri [234]	2012	Saudi Arabia	NPC1	rs1805081 and rs1788799	644 T2D patients 824 healthy controls	
Shalaby [237]	2017	Egypt	GIPR and GLP1R	rs2302382, rs1800437, and rs367543060	150 T2D patients 150 healthy controls	
Ghaedi [244]	2018	Iran	H19 and MEG3	rs217727, rs3741219, and rs7158663	496 T2D patients 473 healthy controls	OR = 1.1 OR = 1.53 OR = 1.79 OR = 1.72
El-Lebedy [255]	2018	Egypt	ANGPTL8, CETP, and NOS3	rs2278426, rs708272, and rs1799983	136 T2D patients 136 healthy controls	
Ghasemi [256]	2019	Iran	ANGPTL8	rs2278426 and rs892066	150 T2D patients 138 healthy controls	OR = 2.41
Hatefi [257]	2018	Iran	miR-410	rs13702	102 T2D patients 98 healthy controls	OR = 1.729 OR = 3.28
Mansouri [260]	2018	Iran	FADS1 and FADS2	rs174537 and rs174575	50 T2D patients 50 healthy controls	
Nuglozeh [265]	2019	Saudi Arabia	PCSK9	L10 Ins, A56V, I474V, and E670G	88 patients 10 healthy controls	
Saravani [268]	2017	Iran	Perilipin and FTO	rs1052700 and rs3751812	183 T2D patients 174 healthy controls	
Galavi [270]	2018	Iran	SREBF-2	rs1052717G/A, rs2267439C/T, and rs2267443G/A	250 T2D patients 250 healthy controls	
Mahrooz [275]	2016	Iran	APOA5	rs662799	161 T2D patients 58 healthy controls	
Alharbi [276]	2014	Saudi Arabia	APOE	rs429358 and rs7412	438 T2D patients 460 healthy controls	OR = 4.39
Alharbi [282]	2015	Saudi Arabia	APOC3	3238 C>G	268 T2D patients 255 healthy controls	
Garme [284]	2018	Iran	NOS2	rs2779248 T/C and rs1137933 C/T	152 T2D patients 157 healthy controls	
Garme [285]	2017	Iran	NOS3	rs1800779	250 T2D patients 250 healthy controls	OR = 0.527 OR = 0.569
Mehrab-Mohseni [288]	2011	Iran	eNOS	VNTR	220 T2D patients 96 healthy controls	OR = 2.0 OR = 2.1 OR = 1.8 OR = 2.6 OR = 2.8
Rahimi [291]	2013	Iran	eNOS	4a/b and G894T	173 T2D patients 101 healthy subjects	
Moguib [292]	2017	Egypt	eNOS	T786C and G894T	200 T2D patients 100 healthy controls	
El-Din Bessa [294]	2011	Egypt	eNOS	Glu298Asp	80 T2D patients 20 healthy controls	
Shoukry [295]	2012	Egypt	eNOS	894G>T, -786T>C, and 27-bp-VNTR	200 T2D patients with nephropathy 200 T2D patients without nephropathy	
Vatankhah Yazdi [306]	2020	Iran	SLC30A8, CDKAL1, TCF7L2, KCNQ1, and IGF2BP2	rs13266634, rs10946398, rs7903146, rs2237892, and rs1470579	162 T2D patients 106 healthy controls	
Shokouhi [307]	2014	Iran	TCF7L2	rs7903146, rs12255372, and rs290487	173 T2D patients 173 healthy controls	OR = 1.98 OR = 3.54 OR = 2.16 OR = 2.23 OR = 4.25 OR = 2.2 OR = 2.24 OR = 2.25
Alami [308]	2012	Iran	TCF7L2	rs12255372 (G/T)	236 T2D patients 255 healthy controls	OR = 1.458 OR = 2.038 OR = 1.52 OR = 1.74
El-Lebedy [309]	2016	Egypt	TCF7L2 and CDKAL1	rs7903146, rs12255372 and rs7756992	180 T2D patients 210 healthy controls	
Ereqat [310]	2010	Palestine	TCF7L2	rs7903146	219 T2D patients 114 healthy controls	OR = 3.34
Erkoç Kaya [311]	2017	Turkey	TCF7L2	rs7903146 and rs12255372	169 T2D patients 119 healthy controls	OR = 1.9 OR = 2.1
Khan [312]	2021	Emirate	TCF7L2	rs4506565 and rs12255372	890 T2D patients 686 healthy controls	OR = 1.16
Saadi [313]	2008	Emirate	TCF7L2	rs12255372 and rs7903146	95 T2D patients 188 healthy controls	OR = 1.47 OR = 1.16
Palizban [314]	2019	Iran	TCF7L2	rs7903146	93 T2D patients 53 healthy controls	OR = 6.035 OR = 3.082
Galavi [317]	2019	Iran	HHEX	rs1111875G/A, rs7923837A/G, and rs5015480 C/T	250 T2D patients 250 healthy controls	
Mansoori [318]	2015	Iran	HHEX and CDKN2A/B	rs1111875A/G and rs10811661C/T	140 T2D patients 140 healthy controls	OR = 1.729 OR = 2.921 OR = 0.237
Torkamandi [324]	2016	Iran	MAP3K1	rs10461617	177 T2D patients 165 healthy controls	OR = 1.44
El-Sherbini [327]	2013	Egypt	TGF-β1	T869C and G915C	99 T2D patients 98 healthy controls	
Saravani [331]	2017	Iran	COL4A3, MMP-9, and TIMP-1	rs55703767, rs17576, and rs6609533	120 T2D patients 120 healthy controls	OR = 0.235 OR = 0.592 OR = 2.429 OR = 2.176
Kassem [335]	2020	Egypt	SERPINB1	rs114597282 and rs15286	98 T2D patients 62 healthy controls	
Zaharna [339]	2010	Palestine	Calpain-10	− 44, − 43, − 63, and del/ins-19	48 T2D patients 48 healthy controls	
Demirci [340]	2008	Turkey	Calpain 10	− 19, − 44, and − 63	202 T2D patients 80 healthy controls	
Mehrabzadeh [342]	2015	Iran	ELMO1	rs741301 and rs1345365	200 T2D patients 100 healthy controls	OR = 1.7 OR = 2.5
Bayoumy [343]	2020	Egypt	ELMO1	rs741301	400 diabetic patients 100 healthy controls	OR = 2.7
Al-Daghri [344]	2016	Saudi Arabia	Snap25	rs363039, rs363043, and rs363050	489 T2D patients 530 controls	
Asgarbeik [350]	2019	Iran	ERRFI1	+ 808 T/G (rs377349)	204 T2D patients 106 healthy controls	
Zarouk [356]	2012	Egypt	ACE	I/D	24 T2D patients 21 healthy controls	
Assali [353]	2011	Iran	AT(1)R/A1166C	A1166C	164 diabetic patients with coronary artery disease (CAD) 145 CAD patients without diabetes	
Al-Saikhan [357]	2017	Saudi Arabia	ACE	I/D	70 T2D 54 T2D with HTN patients 48 healthy controls	
Al-Harbi [358]	2013	Bahrain	MTHFR and ACE	C677T and I/D	171 T2D patients 188 healthy controls	

### Inflammation and immune response

Chronic inflammation associated with T2DM might happen due to the disturbance of anti-inflammatory response [18–20]. Cytokines have an important function in immune reaction that causes the failure of β cell function [21, 22]. Pro-inflammatory cytokines regulate the activity, proliferation, and viability of β-cell [23]. IL-1 family contains three important members: IL-1α and IL-1β as the agonists, and IL1-Ra as the antagonist. There was an association between IL1-Ra polymorphism and T1DM, in which the frequency of (A2) allele and (A1/A2) genotype was significantly higher among diabetics compared with controls in a subpopulation of Saudi cases [24]. IL-4 is involved in regulation of apoptosis and cell proliferation in Th1 cells [25]. IL-4 prevents macrophages from producing pro-inflammatory cytokines such as TNF-alpha and IL-6. A positive association between heterozygous CT variants of the IL-13-1112 and IL-4-590 polymorphisms was observed among Egyptian T2DM cases. In contrast, the homozygous CC genotypes were protective [26]. IL-4 VNTR polymorphism was assessed in Egyptian T2DM cases that showed there was a significant correlation between (A2A2) genotype and increased T2DM susceptibility. There was also a significant reduction in the (A2) allele compared with (A1) in both cases and control group [27]. Serum levels of inflammatory cytokines are increased in T2DM [28–30]. There was significant different frequency of IL-4 -590 genotypes and alleles between Iranian type 2 diabetic cases with nephropathy and healthy controls [31]. As an inhibitory cytokine of autoimmunity and inflammation, IL-10 is involved in the pathogenesis of T2D and its nephropathic complications [32]. It has been reported that there were significant different IL-10-592 genotypes and alleles between T2D cases with and without nephropathy compared with healthy controls. The C/C genotype was correlated with T2D and could be considered as a risk factor among a subpopulation of Iranian subjects [33]. A significant correlation was also observed between IL-10 (− 1082G/A) polymorphism and T2DM susceptibility in Turkish subjects [34]. IL-12B is a critical cytokine for the lymphocytes activation that can be associated with T2D progression. The A/A genotype and A allele of IL-12B3́ UTR A-C polymorphism were correlated with T2D pathogenesis among Iranian subjects [35]. Insulin-secreting cell damage is mediated by the auto reactive Th1 cells. The production of IFN-γ from immune-competent cells is synergistically induced by IL-12 and IL-18 that promote Th1 responses. IL-18 also up-regulates the TNF-α and IL-1 that result in β-cell death [36, 37]. A study was conducted in a subpopulation of Iranian cases to examine any possible correlation between polymorphisms at − 607 and − 137 positions of the IL-18 promoter and susceptibility to type 1 diabetes. There was a significant different frequency of IL-18-137 (C/G) genotypes between subjects who were older than 15 years and controls [38].

CTLA4 belongs to the immunoglobulin family that has a key role in T1D [39]. It has been reported that there were significant different frequencies of G allele and GG genotype of CTLA4+49A>G polymorphism in a sample of Iranian T1D patients compared with controls [40, 41]. The − 1722 (T/C), − 318 (C/T), and + 49(G/A) polymorphisms of the CTLA4 were also examined in Iranian T2D patients. There was a positive correlation between T2D and − 318 C/T and + 49 G/G genotypes, while + 49 A/A and − 318 C/C genotypes were inversely associated with T2D [42]. The A49G polymorphism of CTLA-4 was also assessed among Lebanese diabetic cases that showed a significant higher frequency of the G allele among patients compared with the controls [43].

TNF-α is a cytokine involved in systemic inflammation [44]. Plasma levels of TNF-a are associated with various risk factors of diabetes such as dyslipidemia, obesity, and inflammation [45]. It has a fundamental role in beta cells destruction which is mediated by immune cells. However, TNF-α is inhibited by IL-6 that has protective roles [46]. A significant higher prevalence of IL-1Ra A1A1 and TNF-a2308 AA genotypes, and the subsequent higher prevalence of IL-1Ra A1 and TNF-a2308 A alleles were observed in Egyptian T1D patients. There were also significant lower prevalence of IL-1Ra A1A2 and TNF-a2308 GA genotypes [47]. The -308 G/A polymorphism of TNF-α was also correlated with T2DM and T1D susceptibility among Iranian and Saudi cases, respectively [48, 49]. Regarding a previous study on Egyptian cases, the level of TNF-α was positively associated with total cholesterol, LDL-C, FBG, HbA1c, and creatinine in patients with diabetic foot. Moreover, the levels of circulating TNF-α were three- to fourfold higher in diabetic patients compared with healthy controls. C allele of TNF-α − 1031 T/C was associated with a significant risk for diabetic nephropathy progression. There was an increased TNF-α serum level in patients with diabetic foot who had CC genotype compared with those with TT genotype. Generally, carriers of C allele of TNF-α-1031 T/C had significantly increased risk of diabetic nephropathy [50]. TNF-a G-308A polymorphism in promoter sequence might be involved in glycemic control among Jordanian T2D patients. Poor glycemic control in patients who have − 308GG genotype might be due to the insulin resistance, which is subsequently developed by the high circulating levels of TNF-a protein [51]. PTPN-22 is a member of non-receptor tyrosine phosphatases which is produced by different immune cells [52]. PTPN-22 is inhibitor of effector/ memory T-cell pool required to preserve the balance in immune system [53]. There was a correlation between PTPN-22 1858 T polymorphism and T1D susceptibility among Saudi individuals [54]. PTPN22 SNPs (rs12760457, rs1310182, rs1217414, rs33996649, and rs2476601) were correlated with T1D among Iranians [55]. IL2AR and PTPN22 have pivotal roles in regulation of T-cell activation and tolerance against the self-antigens. IL2RA expression on regulatory T cells is important for its ability to inhibit the immune responses of T cells to tumor antigens, alloantigen, and self-antigens [56]. There was a poor association between T1D susceptibility and T allele of IL2RA (rs11594656) and PTPN22 (rs2476601) polymorphisms in Egyptian children. PTPN22 C1858T polymorphism had potential effect in the early age of onset in female group. T allele of IL2RA and TT genotype increased T1D progression [57].

Adipokines are involved in progression of insulin resistance [58, 59]. Adiponectin (ADIPOQ) belongs to the cytokine family which is associated with insulin-sensitizing and anti-inflammatory properties [60]. There was an association between rs17300539 allele A and increased risk of T2DM among a sample of Iranian patients [61]. The existence of G allele at position − 11,377 and lack of A allele at position − 11,391 also increased the incidence of T1DM among Iranians [62]. There was also higher frequency of TG GG genotype and G allele of adiponectin SNP45 in Iranian obese T2DM cases compared with non-diabetic cases [63]. There was higher frequencies of GT/GG genotype and G allele of ADIPOQ + 45 T>G (rs2241766) in Iranian GDM patients compared with controls [64]. Both TG and GG genotypes of ADIPOQ 45 polymorphism were significantly correlated with T2DM susceptibility. AA genotype of ADIPOQ − 11,391 was also significantly correlated with T2DM susceptibility. Moreover, TT genotype and T allele of ADIPOQ 276 polymorphism were significantly protective among Egyptian subjects [65].

Resistin (RETN) is a cysteine-rich polypeptide produced by adipocytes, immune cells, and endothelial cells [66]. It has been reported that the GG genotype and the G allele of RETN-420C/G was correlated with GDM susceptibility in a sample of Iranian cases [67]. There was increased frequency of RETN + 299 AA in obese cases compared with controls among Egyptian cases. There were also higher GG genotype and G allele frequencies of RETN − 420 in obese patients compared with control group [68]. There were higher frequencies of RETN − 420G/G genotype in Egyptian diabetic patients compared with control group. This prevalence was much more in diabetic patients who suffered from CVD compared with patients without CVD [69]. Chemerin is an adipokine involved in the regulation of adipogenesis and glucose metabolism [70, 71]. A correlation between chemerin rs4721 polymorphism and the risk of GDM has been reported among Iranian cases in which GG genotype and G allele were more frequent in non-GDM group compared with GDM group. Moreover, GT and GT + TT genotypes were correlated with a higher risk of GDM progression compared with GG genotype [72]. Omentin is one of the most important visceral fat adipokines [73]. It has been shown that Omentin V109D polymorphism was correlated with insulin resistance and familial history of diabetes among Iranian T2D patients [74]. Irisin is a cytokine that regulates energy metabolism by conversion of white into brown adipose tissue [75]. It is produced by the FNDC5 cleavage [76]. A study suggested a correlation between reduced T2DM susceptibility and the FNDC5 rs16835198 TT genotype among Egyptian cases. There was also correlation between the G allele and HOMA-IR and elevated fasting insulin. Decreased circulating levels of irisin was significantly associated with nephropathy in T2DM patients [77].

Macrophage migration inhibitory factor (MIF) as a T cell-derived pro-inflammatory cytokine prevents the macrophages migration which is the regulator of cellular inflammation [78]. A study was done to examine whether MIF expression level and the MIF173 G>C genotype distribution are different in both men and women of Iranian T2DM patients with or without depressive symptoms. C allele was reported to be correlated with susceptibility to depression in female T2DM subjects [79]. CD226 is an immunoglobulin-like transmembrane glycoprotein expressed on monocytes and NK cells [80, 81]. It has been reported that there was a correlation between CD226 rs763361 C<T polymorphism and susceptibility to T1D in Egyptian children. The onset of diabetes was significantly observed at a younger age in patients, who had T allele and TT genotype. The frequency of T allele was significantly higher in patients whose diabetes started at age 10 years [82].

Human leukocyte antigen-G (HLA-G) is associated with reduced immune response to protect the fetus from immune rejection or avoid allograft rejection in organ transplant patients [83, 84]. HLA-G disrupts the cytotoxic function of CD8+ T and NK cells and the maturity of dendritic cells [85, 86]. It is associated with autoimmune disorders including T1DM in which activated T cells cause the destruction of β cells during immune response [87–89]. Human leukocyte antigen is involved in self-/non-self-recognition that contains class I (HLA-A-C), class II (HLA-DP, -DQ and -DR), and class III loci [90, 91]. The occurrence of DQA1*0501, DRB1*0301, and DQB1*0201 alleles and their haplotypes were evaluated in Iranian T1D subjects. All three alleles were correlated with T1D. The patients had a higher frequency of DRB1*0301 allele [92].

### Nuclear receptors

Various studies have been suggested that the prevalence of serum vitamin D deficiency is high all around the world [93–97]. Vitamin D has a pivotal role in bone metabolism and also functions as an antioxidant, anti-angiogenic, and anti-proliferator factor. It is also involved in several diseases such as diabetes, metabolic syndrome, obesity, and osteoporosis [98–103]. Vitamin D deficiency triggers autoimmune destruction of ß -cells that initiates T1DM through the loss of immunomodulation [104, 105]. It affects target tissues via its receptor called vitamin D receptor (VDR), which belongs to the nuclear receptor protein family. Vitamin D is correlated with macrophage activation, maturation of antigen-presenting cells, and inhibiting dendritic cell differentiation [106]. It prevents T-cell activation and TNF-α, IL-1, IL-12, and IFN-c productions [107, 108]. VDR polymorphism and vitamin D levels are involved in T1DM progression in which high vitamin D levels are protective for ß-cells [109]. The correlation between VDR gene polymorphisms at four positions (FokI, BsmI, TaqI, and ApaI) and T1DM was investigated among a subpopulation of Iranian subjects. It has been reported that there was significant higher frequency of TaqI-T allele in healthy controls compared with TIDM subjects. FokI-F allele increased risk of T1D, while T allele seemed to be protective in the TaqI polymorphism [110]. The correlation between GDM susceptibility and VDR ApaI/TaqI polymorphisms was investigated among Iranian cases. There was a significant different genotype frequency between GDM and non-GDM pregnant women. CC genotype was more frequent in GDM groups. Compared to AA genotype, CC genotype carriers had significantly increased risk of GDM progression. ApaI polymorphism was demonstrated to be correlated with GDM. There was also a correlation between TaqI polymorphism and the onset of GDM. Accordingly, the TT genotype carriers had a remarkably higher risk for GDM compared with TC genotype carriers. The TaqI-T allele carriers were more likely to develop GDM than cases with C alleles [111]. A case–control study was performed among Iranian type 2 diabetic subjects to investigate the correlation between VDR gene polymorphism (rs7975232 C>A, rs731236 T>C, and rs4516035 T>C) and risk of DN. There were significant higher frequencies of CCC, TCC haplotypes in DN group [112]. There was a significant association between FokI VDR genotype variations and an increased risk of GDM among Iranians [113]. There were different frequencies of the FokI and BsmI VDR genotypes between Egyptian T1DM and controls. The frequency of VDR Bb genotype, bb genotype, and b allele in T1DM patients was significantly higher than in control individuals. T1DM cases had also significant higher frequency of Ff and ff genotypes and f allele compared with controls [114]. There were also significant correlations between ApaI and BsmI allele and genotype distributions and an increased T1DM susceptibility among Egyptian cases [115]. It has been shown that the interaction between HLA and VDR alleles was interceded by the vitamin D response element (VDRE) in the promoter of some HLA-DRB1 alleles that was involved in pathogenesis of T1DM [116]. The associations between VDR SNPs and HLA alleles were assessed among Saudi T2DM patients. BsmI and TaqI Polymorphisms of the VDR gene were significantly correlated with susceptibility to T2DM. A higher expression of VDR was also observed in TaqI (AG) and BsmI (CT) genotypes, which were more commonly found among T2DM cases. The presence of HLA-DRB*04 with such VDR SNPs increased T2DM susceptibility among Saudi patients [117]. The CT genotype and C allele of FokI were correlated with reduced diabetes susceptibility among Saudi subjects [118]. There was a positive correlation between T1DM and BsmI and FokI polymorphisms in Saudi children [119]. VDR gene FokI SNPs were correlated with GDM among Turkish pregnant women independently. The prevalence of the VDR gene FokI CT and TT genotype was higher among individuals with GDM than the non-GDM controls [120]. VDR gene FokI polymorphism was significantly correlated with T1DM among Kuwaiti Arab children. There was also a positive correlation between T1DM and the C allele TaqI polymorphism was found [121]. Low serum levels of 1,25(OH)2D3 and 25(OH)D3 are associated with impaired function of immune system and T1D susceptibility [122]. The CYP2R1 catalyzes vitamin D3 to D 25-hydroxyvitamin D3 (25(OH) D3). CYP27B1 catalyzes the 25(OH)D3 to 1,25(OH)2D3 in renal cells [123]. It has been reported that CYP2R1 GG or CYP27B1 CC genotype carriers were associated with the increased type 1 diabetes progression. There was increased risk of type 1 diabetes for the GG genotype of CYP2R1 among subjects with CC genotype of CYP27B1. There was higher frequency of CYP2R1 GG+CYP27B1 CC among patients compared with healthy controls. The frequency of CYP2R1 GG carriers was also lower among CA/AA patients compared with CYP27B1 CC patients [124].

Sex hormones may significantly be associated with diabetes mellitus. It has been reported that estrogen can regulate calcium signals, insulin secretion, and K-ATP channel activity [125, 126]. Estrogens are involved in the stimulation of insulin synthesis in pancreas β-cells, prevention of β-cell apoptosis, increasing hepatic insulin sensitivity, and improvement of insulin action in skeletal muscles [127]. Estrogen functions via estrogen receptor (ER) in which estrogen binding with ERs regulate the expression of target genes [128]. A study was done to discover the correlation between PvuII and XbaI polymorphisms and T2DM among Iranian cases. It has been reported that there was a remarkable correlation between both PvuII and XbaI polymorphisms of ERα and T2DM susceptibility. PvuII and XbaI polymorphisms in ERα were also increased with aging [129]. There was also a correlation between the PvuII and XbaI variants and T2D among Iranian males. The frequencies of T allele of PvuII and A allele of XbaI polymorphisms were remarkably higher in male T2D cases compared with controls. Moreover, normal males carrying the AA genotype of PvuII polymorphism had increased levels of fasting glucose [130]. ESR1 positively influences GLUT4 expression and insulin signaling in skeletal muscle. It is suggested that stimulation of estrogen receptor with propylpyrazoletriyl (an agonist of the receptor) in skeletal muscles leads to a higher insulin-stimulated glucose uptake [131]. There was a correlation between ESR1 PvuII variant and T2DM susceptibility in Palestinian cases [132]. Sex hormone-binding globulin (SHBG) has been considered as one of the environmental and genetic factors that have a role in the pathophysiology of type 2 diabetes [133]. It is negatively correlated with insulin levels that cause T2DM progression. It has been reported that SHBG down-regulation increased the estradiol to regulate glucose metabolism among Egyptian T2DM cases. The rs6257 allele carriers were correlated with SHBG down-regulation, while the SHBG up-regulation was observed in rs6259 allele carriers [134].

Peroxisome proliferator-activated receptor-γ (PPAR-γ) belongs to the nuclear receptor protein family associated with regulation of metabolic processes [135, 136]. It regulates adiponectin and leptin expressions that have pivotal roles in insulin sensitivity of skeletal muscles [137–139]. Association between Pro12Ala PPAR-γ-2 variant and insulin resistance was assessed in a sample of Iranian cases. There was lower frequency of Ala allele in diabetic patients compared with controls. There were also reduced fasting insulin levels in Ala/Ala and Pro/Ala compared with in Pro/Pro carriers in control cases [140]. PGC-1α encodes an inducible transcriptional co-activator that interacts with PPAR-γ to elevate glucose uptake in muscle cells and also regulates T2D-associated metabolic processes, including hepatic gluconeogenesis and insulin release by the beta cells [141]. It has been shown that there were significant different frequencies of A allele of Thr528Thr and Gly482Ser variants between patients and healthy controls. 394-GG/482-GA/528-GG haplotypes of PGC-1alpha were also remarkably correlated with a higher risk of T2D in a subpopulation of Iranian cases [142]. PPAR-c is involved in subcellular metabolism of arterial wall macrophage and formation of adipose tissue [143, 144]. Moreover, it regulates the insulin sensitivity and induces the transcription of adipocyte-specific genes which are involved in the fatty acid uptake, glucose uptake, and insulin signaling [143]. PPAR-c Pro12Ala polymorphism (rs1801282) was more prevalent among Egyptian diabetic patients suffering from coronary artery disease (CAD) complications compared with patients without any complications. This finding suggested an increased risk of CAD in diabetic patients with PPAR-c Pro12Ala polymorphism. Moreover, diabetic patients with PPAR-c Pro12Ala polymorphism who suffered from CAD complications had higher cholesterol and LDL levels compared with others [145].

### Stress response and detoxification

Glutathione S-transferases (GST) are a family of phase II metabolic enzymes which protect cells from oxidative damage through detoxification of carcinogenic and toxic compounds by glutathione conjugation [146–150]. GDM progression and its complications are associated with oxidation and antioxidant imbalance which is caused by increased levels of circulating reactive oxygen species (ROS) and deregulation of anti-oxidative enzymes [151]. A study was done to examine the correlation between GSTM1 and GSTT1 polymorphisms and GDM susceptibility in a sample of Iranian cases. GSTM1 null genotype was involved in increased GDM susceptibility [152]. A significant increased frequency of GSTM1-null genotype was reported in Iranian T2DM subjects compared with controls [153, 154]. A higher frequency of GSTT1 null genotype was also observed in Egyptian T1DM patients compared with healthy controls [155]. There were significant higher frequencies of GSTT1 and GSTM1 polymorphisms in Egyptian T2DM cases compared with controls [156]. Role of GST-P1 (Ile105Val) polymorphism was assessed in Egyptian T2DM cases and controls. There was higher G allele frequency in T2DM patients compared with healthy controls. There were also significant different frequencies of the Ile/Val genotype between patients and the controls [157]. It has been shown that GSTT1 rs17856199-C was significantly correlated with T2DM risk among Egyptian cases. CC homozygote carriers had higher risk of T2DM progression compared with non-carriers [158]. About 15% of liver transplant recipients may reveal signs of post-transplant diabetes mellitus or New-onset diabetes mellitus (NODM) which is a metabolic disease without any previous history of hyperglycemia [159, 160]. Increased plasma glucose and FFA oxidation and also ROS generation through the respiratory electron transport chain are as a result of elevated plasma free fatty acids (FFAs) and post-transplant hyperglycemia [161, 162]. It has been reported that GSTP1 genotypes were significantly associated with risk of NODM progression. The heterozygous (AG) genotype was more frequent in liver transplant cases with NODM compared with non-NODM cases. AG allele of the GSTP1 (A313G) increased risk of NODM in a subpopulation of Iranian cases [163]. A study evaluated the genotype frequencies of the GSTP1, GSTT1, and GSTM1 polymorphisms in order to find the probable correlation of the GST polymorphisms with susceptibility to DM among the Turkish individuals. There was a significant different frequency of the GSTM1 null mutations between diabetics and the controls. Susceptibility to DM was higher 4 times in patients with a combination of the GSTT1 positive genotype and GSTM1 null genotype and the GSTP1 Val allele [164]. Base excision repair (BER) is involved in DNA repair of oxidized bases [165]. The first stage of the BER pathway is the identification and removal of the altered base (8-OHdG) with the help of OGG1. This enzyme is involved in cleavage of the glycosylic bond between the sugar moiety and the modified base, which leads to an apurinic/apyrimidinic (AP) site in DNA. It has been reported that OGG1 (H+M) and GSTT1 null genotypes significantly increased the T2DM susceptibility among Turkish cases. Four times higher risk of having T2DM was identified among subjects who were carriers of the combined GSTT1 null, GSTM1 null, and GSTP1 (H+M) genotypes [166].

Superoxide dismutase (SOD) catalyzes the conversion of superoxide radical into oxygen and hydrogen peroxide. Hydrogen peroxide is also degraded by catalase. Therefore, SOD and catalase are pivotal antioxidant agents in living cells exposed to oxygen. A research among Egyptian population has showed the correlation between SOD + 35A/C and CAT 1167C/T polymorphisms and T2DM. They revealed the association of CAT-C1167T polymorphism with diabetes susceptibility in which the heterozygote CT genotype was significantly more frequent in patients compared with healthy individuals. There was significant increased T allele frequency in patients compared with healthy controls. Considering the polymorphism of + 35A/C SOD1, homozygote CC genotype carriers had higher T2DM susceptibility compared with AA genotype carriers [167]. Microsomal epoxide hydrolase (mEPHX) has important roles in epoxide metabolism. Microsomal (EPHX1) and soluble (EPHX2) epoxide hydrolases are involved in regulation of the oxidation status of lipid- and xenobiotic-derived substrates. A research among Egyptians has been suggested the relation of mEPHX1 (rs1051740) polymorphism with T2DM susceptibility. The Tyr113/Tyr113 was the most frequent genotype in the healthy controls compared with patients. There was decreased risk of T2DM among individuals with homozygous wild genotype. Lower insulin sensitivity and higher fasting insulin levels were also observed among the His139/His139 genotype carriers comparing with Arg139 allele carriers [168]. It has been also reported that EPHX2 rs751141 A allele was protective against diabetic nephropathy among Egyptian T2DM cases [169].

Heat-shock proteins (HSPs) are chaperones produced during ischemia, heat shock, and stressful conditions. JAK/STAT pathway is involved in oxidative stress adaptation via HSP70 regulation [170]. There were significant increased frequencies of CC, AC, and AA genotypes (− 110 A/C HSP 70) in nephropathic T2DM, non-nephropathic T2DM, and control groups, respectively. The C allele of (− 110 A/C HSP 70-1) polymorphism was involved in nephropathy in diabetic patients. There were significant different prevalence of CGGT, CCGT, AGGT, and AGAT haplotypes between diabetic patients suffering from nephropathy and the control group [171]. N-acetyltransferase 1 (NAT1) and NAT2 are two families of enzymes involved in catalyzing the acetylation of several heterocyclic and aromatic amine carcinogens as well as several hydrazine and aromatic drugs [172, 173]. NAT2 is capable of O-acetylation and N-acetylation that are involved in xenobiotics detoxification [174]. The rs1799931 G>A polymorphism of NAT was significantly different between the Saudi T2DM patients and healthy controls [175].

Enzymatic antioxidants including paraoxonase 1 (PON1), catalase (CAT), SOD, and glutathione peroxidase (GPx) are the main components of the antioxidant system [176, 177]. PON1 is a glycoprotein belongs to the hydrolase family that is involved in inhibition of LDL oxidation and peroxidation [178]. There was a remarkable correlation between PON1 activity and polymorphism − 108C>T in Iranian T2D subjects in which TT genotype carriers had the lowest PON1 activity [179, 180]. There was a significant correlation between PON1 promoter polymorphism (− 108C>T) and its Arylesterase-based activity in Iranian T2DM cases compared with controls [181]. There were correlations between L55M and PON1 Q192R polymorphisms and T2DM among Egyptian cases. Diabetic patients had significant lower serum concentration of the PON1 enzyme compared with controls in which the lowest concentrations were related to the 192R allele [182]. R allele of PON1 Q192R polymorphism also increased GDM susceptibility among Saudi individuals [183]. The correlation between PON1 55 leucine (L)/ methionine (M) and CAT-262 cysteine (C)/threonine (T) genetic polymorphisms and the level of malondialdehyde were assessed among Turkish T2DM cases. CAT antioxidant enzyme activity was significantly lower in carriers of TT genotype compared with the CT genotype among diabetic patients. The activity of PON1 was lower in carriers of MM genotype comparing with carriers of LL genotype among diabetic patients and controls [184]. Myeloperoxidase (MPO) belongs to the heme peroxidase superfamily that produces various diffusible radical species and reactive oxidants to initiate peroxidation of lipids [185–187]. A study evaluated the associations between T2DM and MPO-463G/A polymorphism among Turkish cases. GG genotype was more frequent among patients than the controls. However, the rate of carrying the A allele and the AA genotype for non-diabetic individuals was higher compared with diabetic patients [188].

### Insulin signaling and transporters

Insulin promotes a wide range of growth and metabolic effects through insulin receptor binding and tyrosine kinase activation that phosphorylates insulin receptor substrate protein 1 (IRS1) (189). It has been reported that the A allele of IRS1‐rs10498210 G/A polymorphism induced risk of type 2 diabetes among Iranians [190, 191]. There were significant higher frequencies of D allele (IRS-2 Gly1057Asp) and of R allele (IRS-1 Gly972Arg) polymorphisms in Iranian T2DM cases compared with controls. Normal cases carrying the GD+DD genotypes of IRS-2 Gly1057Asp had remarkably increased fasting plasma glucose and cholesterol in comparison with GG genotype carriers [192]. It has been observed that the IRS-1(Gly972Arg) AA and GA were the most frequent genotypes in Egyptian T2DM patients. Arg 972 IRS-1 polymorphism was involved in inhibiting of IRS-1/PI3-kinase/Akt axis. A allele and GA, GA+AA genotypes had significant higher frequencies in T2DM compared control cases. IRS1 (r.2963G>A) polymorphism was an efficient determinant for insulin resistance in T2DM patients [193]. IRS-2 DD genotype of G1057D polymorphism had a higher prevalence among Turkish GDM patients compared with control group. IRS-2 DD genotype was accompanied by a 2.97-fold risk for GDM. Carriers of the D allele had also a significant higher risk for GDM [194]. The association of IRS-1 G972R polymorphism and GDM was assessed among the Saudi women population. There was significant correlation between allele Arg972 of the IRS-1 and GDM. G972R homozygosity also increased risk of GDM among Saudi women [195]. IGF2BP2 is an mRNA-binding protein involved in regulation of IGF2 protein modifications [196]. It has been revealed that both rs4402960 minor (T) allele containing haplotypes (TA and TC) of IGF2BP2 were correlated with T2DM susceptibility in a sample of Lebanese cases [197]. Src-homology-2 B adaptor protein 1 (SH2B1) is a positive regulator of insulin receptor [198, 199]. A study revealed that the rs4788102 of SH2B1 was significantly correlated with GDM among Saudi cases [200].

The insulin secretion pathway begins with the prevention of ATP-sensitive potassium channels by glucose, β-cell membrane depolarization, and increased intracellular calcium concentration that stimulate exocytosis of insulin-containing granules. This channel includes ABCC8 and KCNJ11 subunits [201]. The KCNJ11 (rs5219) was examined to explore the correlation between E23K polymorphism and T2DM susceptibility among Iranian cases. There was a correlation between E23K polymorphism and T2DM in which K allele carriers had higher risk of disease [202]. It has been shown that the KCNJ11 rs5219 polymorphism was a risk factor for T2DM among Syrian cases [203]. Alfa2-Heremans-Schmid glycoprotein (AHSG) is a serum glycoprotein involved in immune response, bone metabolism, and insulin resistance [204–207]. It has a role in insulin resistance by inhibition of insulin receptor phosphorylation [208]. A study evaluated the association between 767G>C polymorphism of AHSG and GDM susceptibility among Turkish population that showed the homozygous GG variant might have protective effects against GDM [209]. Selenoprotein P (SEPP1) is mainly produced by liver and is involved in the transport of Selenium from the liver to other organs [210]. The rs13154178 polymorphism was more frequent among Turkish GDM group compared with the controls [211]. Glut1 encoded by SLC2A1 is a carrier protein that preserves the normal glucose concentration and uptake required to maintain respiration in all cells [212]. SLC2A1 HaeIII polymorphism was examined in a subpopulation of Iranian T2DM cases in which CC was detected to be the most common genotype for SLC2A1 HaeIII polymorphism. The frequency of CC genotype was also higher in the DN group, and this genotype was significantly associated with the risk of DN. Therefore, C allele of HaeIII was proposed to be a strong risk factor for the T2DM-related DN progression [213]. The correlation between SLC30A8 rs13266634 polymorphism and T2D was examined in a subpopulation of Iranian patients. There was a significant correlation between rs13266634 polymorphism and T2D in which cases with TT genotype had lower OR compared with CC and CT genotypes. The cases with C allele had higher OR compared to those with allele T [214]. SLC30A8 encodes a zinc transporter which is essential for insulin’s storage, secretion, and stability in the beta cells [215]. It regulates the entry of zinc ions into insulin secretory vesicles from the cytoplasm, where zinc ions prevent insulin degradation by stabilizing insulin hexamers [216]. It has been reported that CC genotype of SLC30A8-rs13266634 polymorphism was significantly associated with the diabetic group in a subpopulation of Jordanian cases [217]. The rs13266634 polymorphism of SLC30A8 was significantly correlated with an increased risk of T2DM in Saudi cases [218].

Organic cation transporters (OCTs) are involved in Metformin transportation. OCT3 regulates the neurotransmission and homeostasis in the central nervous system [219]. It has been reported that the minor allele of OCT3 rs3088442GOA variant was protective toward T2D among Iranian cases. In contrast, rs2292334GOA variant increased risk of T2D. A allele carriers of rs2292334GOA had elevated risk of T2D in obese cases in comparison with non-obese cases [220]. As an endoplasmic reticulum (ER) glycoprotein, Wolferamin (WSF1) is involved in calcium transportation in ER [221, 222]. ER in beta cells had significant influences on the production and secretion of insulin [223–226]. The pathogenic WSF1 variants and epigenetic modifications result in glucose intolerance, and insulin deficiency that causes ER stress and beta cells apoptosis [222, 227, 228]. There was a significant association between rs1801214 and T2DM in a sample of Iranian cases. T allele of rs1801214 and G allele of rs1046320 reduced T2DM susceptibility [229]. Uncoupling protein 2 (UCP2) is an anion carrier protein in inner mitochondrial membrane, involved in energy homeostasis, insulin secretion, and metabolism of lipids [230, 231]. There was a correlation between UCP2-45 bp I/I polymorphism and increased T2DM risk in a sample of Iranian cases [232]. NPC1 encodes a protein with vital roles in the intracellular trafficking of sterols. This large multi-domain protein is located in lysosomes and endosomes and its function is to transport lipids to several cellular compartments [233]. According to its role in the transport of cholesterol, this protein plays a vital role in the metabolism of lipids [233]. A statistically significant correlation was found between T2D and rs1788799 among Saudi cases [234].

### Glucose and insulin metabolism

Incretins are protein hormones secreted by gastrointestinal tract (GIT) due to the food ingestion which are involved in regulation of insulin response [235]. They promote insulin secretion while inhibiting glucagon secretion. Glucose‐dependent insulinotropic polypeptide (GIP) and Glucagon-like peptide-1 (GLP-1) have similar roles like incretins. GLP-1 and GIP bind to particular receptors to activate adenylate cyclase and the subsequent increased level of intracellular cAMP [236]. A correlation between T2DM and GIPR rs2302382 polymorphism has been shown among Egyptian population. There was a correlation between susceptibility to T2DM and A (rs2302382) C (rs1800437) haplotype, while the C (rs2302382) G (rs1800437) haplotype was protective [237].

MicroRNAs (miRNAs) as the pivotal regulators of glucose metabolism and homeostasis are involved in T2DM pathogenesis [238]. Many studies showed that miRNAs are significantly correlated with pancreatic islet development, insulin secretion, and insulin resistance [239]. LncRNAs such as MEG3 and H19 were suggested to adjust β cell function and glucose homeostasis [240–242]. H19 down-regulation was reported in the muscle of both insulin-resistant mice and human diabetic patients [241]. MEG3 was proposed to be associated with the pathogenesis of T2D and its micro vascular complications [243]. H19 rs217727- TT and the AA genotype of MEG3 rs7158663 were reported to be correlated with a significant increased risk of T2D among Iranians [244]. A case–control study was conducted to examine the impact of rs895819 (T/C) miR-27a on T2DM susceptibility among Iranian subjects. The C allele was significantly protective in which CC carriers had decreased T2DM risk compared with TT homozygotes and CT heterozygotes [245].

### Lipid and cholesterol homeostasis and metabolism

Angiopoietin-like proteins (ANGPTLs) have vital roles in lipid metabolism and trafficking. The activity of lipoprotein lipase (LPL) is regulated by ANGPTL8, which is modulated by insulin [246]. ANGPTL8 belongs to the angiopoietin‐like protein family that is mostly expressed in the liver and fat tissue [247]. It promotes β-cell proliferation that subsequently increases islet size and glucose metabolism [248, 249]. ANGPTL8 is associated with T2DM progression, lipid metabolism, and insulin resistance [250, 251]. Cholesteryl ester transfer protein (CETP) has a vital role in HDL-C metabolism. CETP is involved in transferring cholesteryl esters from HDL-C to LDL and VLDL that reduces HDL-C concentration and changes susceptibility to atherosclerotic vascular disorders [252–254]. It has been reported that CETP rs708272 and ANGPTL8 rs2278426 variants were correlated with increased risk of T2DM. T allele was protective against CVD progression, while C allele increased risk of CVD in T2DM patients. The risk of T2DM was increased in homozygous B1 allele carriers in Egyptian T2DM cases [255]. It has been reported that there was a significant different genotype and allele frequencies of ANGPTL8 rs2278426 (C/T) variant between T2DM patients and controls. CT genotype was more susceptible to develop T2D. There were significant higher insulin resistance in CT genotype carriers compared with CC and TT genotype carriers [256]. LPL is a pivotal regulator of body fat saving through eliminating triglycerides (TGs) from blood and transferring to the fat cells. The correlation between LPL rs13702 C/T polymorphism and T2DM was explored in a sample of Iranian population. CC genotype was considerably related to the chance of T2DM. CT genotype was protective against T2DM. The rs13702 C allele damaged the binding sequence of miR-410 and up-regulated LPL which reduced serum triglyceride level and relocates FFA to peripheral tissues to cause insulin resistance [257].

The lipid profile is affected by diet and Fatty acid desaturase 1 (FADS1) and FADS2 alleles [258]. Higher susceptibility to specific metabolic disorders in adulthood is influenced by the amount of total cholesterol, LDL, HDL, and TGs in childhood which is influenced by FADS1 and FADS2 [259]. It has been reported that FADS1 (rs174537) polymorphism had a remarkable correlation with diabetes type 2 among Iranian cases [260]. Low-density lipoprotein receptor (LDLR) is down-regulated by PCSK9, which induces lysosomal degradation of LDLR in both pancreatic and liver cells. PCSK9 down-regulation increases the LDL-C clearance that results in hypocholesterolemia [261]. It has been reported that cell survival, insulin production, and secretion might be impaired by LDLR-mediated entry of excess extracellular LDL-C in beta cells [262–264]. A study in a Saudi Arabia samples has been investigated the prevalence of four common PCSK9 polymorphisms. They showed a prevalence of 29.59% and 35.71% for the E670G and I474V variations, respectively, which were the most common variations. Both E670G and I474V variations were observed in approximately 60% of patients. There was also a correlation between L10ins/ A56V variations and lower plasma cholesterol level [265]. Perilipin (PLIN) is a phosphoprotein target of protein kinase A (PKA). Lipolysis of TAG’s in lipid droplets is mediated through the activation or inhibition of hormone-sensitive lipase (HSL) by phosphorylated and non-phosphorylated perilipin, respectively [266, 267]. There was a correlation between PLIN (rs1052700) polymorphism and T2D in a sample of Iranian cases [268]. Sterol regulatory element-binding transcription factor-2 (SREBF-2) is involved in regulation of cholesterol hemostasis [269]. There was a significant correlation between SREBF-2 rs2267439C/T variant and T2D susceptibility in a subpopulation of Iranian cases [270]. Diabetic nephropathy (DN) is one of the leading causes of morbidity and death in T2D patients and has become a serious health problem [271]. About 30–40 percent of diabetic patients are affected by DN which is a prevalent and important micro vascular diabetic complication [272]. DN is the main reason for end-stage renal disease that is hardly identified with elevated creatinine and proteinuria levels, while reduced glomerular filtration rate. New approaches are needed to develop the diagnosis of devastating complications of diabetes [271]. APOE belongs to the apolipoprotein family of polymorphic glycoproteins involved in cholesterol transport [273]. Apolipoprotein A5 (APOA5) has a pivotal role in TG metabolism [274]. APOA5 up-regulation is associated with reduced TG plasma levels [274]. Correlation between APOA5 (rs662799) variants and lipid profile levels were investigated in case–control study on Iranian T2D patients. Higher TG levels were observed in CC carriers in DN+, DN−, and control groups [275]. APOE e2 and e4 alleles were correlated with the higher risk of T2DM, while e3 was protective against diabetes among the Saudi population [276]. ApoC3, as a natural inhibitor of lipoprotein lipase, is involved in the modulation of the metabolism of triglyceride-rich lipoproteins [277, 278]. The levels of ApoC3 are positively associated with the levels of plasma triglyceride, which might be due to its inhibitory effect on lipoprotein lipase [279–281]. A significant correlation was observed between 3238C>G polymorphism of ApoC3 and susceptibility to T2DM among the Saudi population [282]. It has been revealed that the e3, e4, and e2 alleles were the first three most prevalent alleles of Apo E polymorphism among Turkish diabetic cases. The prevalence of the Apo E4 genotype was lower in normal controls compared with the diabetics with nephropathy [283].

### Nitric oxide

Nitric oxide (NO) is a pivotal regulator of endothelial action and homeostasis that is derived from L-arginine by nitric oxide synthases (NOSs) which are necessary for cellular signaling and insulin secretion. It has been reported that NOS2 rs2779248T/C and rs1137933C/T gene polymorphisms significantly increased T2D risk in a sample of Iranian cases. T allele and CT genotype of NOS2 rs1137933C/T and CC genotype of NOS2 rs2779248T/C were remarkably correlated with increased risk of T2D, while TC genotype of NOS2 rs277 was remarkably protective for T2D [284]. A positive correlation was also observed between the rs1800779 (A/G) polymorphism of NOS3 and T2D in dominant (AG+GG vs. AA) and codominant (AG vs. AA) patterns among Iranian subjects [285]. Endothelial-derived NO is produced by eNOS that is involved in vascular action in insulin and glucose transfer to the skeletal muscles [286]. eNOS regulates insulin secretion and glucose metabolism that can be associated with T2D progression [287]. A remarkable different allele and genotype frequency of eNOS VNTR polymorphism was reported among Iranian diabetic cases. There was a significant correlation between this polymorphism and cases with diabetic neuropathy [288]. The presence of eNOS variants may also cause nephropathy and endothelial disorder via diminished production of NO [289, 290]. The eNOS 4a or 894 T allele increased DN progression in a sample of Iranian T2DM cases [291]. The correlation of type 2 diabetes with TT genotype of eNOS G894T variant was also found among a sample of Egyptian cases [292]. End-stage renal disease (ESRD) is mainly caused by DN. Pathophysiological specifications of DN are an early phase with hyper filtration, glomerular hypertrophy, and microalbuminuria which leads to an advanced phase with proteinuria progressive glomerulosclerosis, and reduced renal function [293]. It has been reported that eNOS polymorphism was involved in ESRD among Egyptian T2D patients in which TT genotype highly increased the ESRD susceptibility. There was significant reduced plasma nitrate/nitrite level and serum NOS activity in TT genotype carriers compared with GG and GT genotypes, mentioning the Glu298Asp polymorphism as an important risk factor of DN to ESRD progression via NO levels reduction [294]. It has been suggested that the T allele and the TT genotype of eNOS 894G>T polymorphism, and the C allele and the CC genotype of − 786 T>C SNP, were significantly more prevalent among Egyptian diabetic patients suffering from nephropathy than those without nephropathy. Serum levels of NO were also significantly reduced in (− 786 T/C) CC and TC genotypes compared with TT genotype, and also 894G>T TT and GT genotypes compared with GG genotype among patients with diabetic nephropathy and patients without diabetic nephropathy [295].

### Signaling pathways

WNT is a pivotal signaling pathway that is involved in regulation of various physiological and pathophysiological processes such as cell cycle, metabolism, apoptosis, immune response, and tumorigenesis [296–299]. Transcription factor 7-like 2 (TCF7L2) belongs to the high mobility group box transcription factors involved in WNT signaling pathway. It regulates cortisol/aldosterone secretion, pancreatic β-cell function, inflammatory status, and preadipocyte differentiation [300]. TCF7L2 is associated with Wnt signaling pathway via regulation of GLP-1, which is involved in blood glucose homeostasis [301, 302]. Deregulation of Wnt signaling has a pivotal role in insulin resistance [303]. T allele of TCF7L2 (rs7903146C/T) polymorphism was considered as a risk allele in diabetes among Iranian cases [304–306]. The correlation between rs12255372, rs7903146, and rs290487 polymorphisms of TCF7L2 and T2DM was investigated among Iranian cases. There were correlations between T allele and genotypes of these variants and T2DM. Normal cases carrying the GT+TT genotypes of the rs12255372 variation had a remarkably higher WHR compared with GG genotype carriers [307]. The correlation between T2DM and TCF7L2 rs12255372 variant was assessed in a subpopulation of Iranian cases in which the minor T allele of TCF7L2 rs12255372 significantly elevated the T2DM risk. There was significant different frequency of TT genotypes in T2DM cases compared with controls [308]. TCF7L2 rs7903146 and rs12255372 were also correlated with T2DM susceptibility among Egyptian population [309]. The rs7903146 variant of TCF7L2 was significantly correlated with T2DM among Palestinian individuals [310]. The rs12255372 G/T substitution and the rs7903146 C/T substitution were considerably correlated with T2DM. The TTTCTT haplotype for rs11196213, rs11196205, rs12255372, rs3814573, rs7901695, and rs7903146 was a risk factor for the occurrence of T2DM among Turkish populations [311]. There were significant correlations between TCF7L2 rs12255372 polymorphism and T2DM in among Emirati cases in which “TT” genotype increased the T2DM risk [312, 313]. Adropin is involved in insulin resistance and glucose oxidation. In a case control study, the serum levels of adropin and rs7903146 polymorphism were examined in Iranian T2DM subjects. Remarkable different frequency of adropin genotypes was observed between subjects and control groups. TT genotype carriers had the highest adropin serum level whereas healthy people with CC genotype had the lowest adropin serum level. The rs7903146T/T and rs7903146C/T genotypes also increased the risk of T2DM [314].

Hematopoietically expressed homeobox (HHEX) is a transcription factor involved in regulation of WNT signaling that has pivotal roles during pancreas development [315, 316]. HHEX variants have been proved to be associated with T2D [316]. The correlation between rs1111875G/A and rs5015480C/T polymorphisms of HHEX and risk of T2D was investigated among Iranian diabetic cases. GA and AA genotypes of rs1111875G/A increased risk of T2D. CT genotype of rs5015480C/T was also significantly associated with T2D progression [317]. It has been found that GG genotype of HHEX rs1111875 A/G polymorphism had an important relationship with T2DM susceptibility among Iranians. GA genotype was also significantly protective in T2DM [318].

MAPK signaling pathway is involved in signal transduction of hyperglycemia [319]. Deregulation of MAPK pathway and related impact on insulin pathway was reported in T2DM patients [320–323]. It has been observed that there was a significant correlation between MAP3K1 (rs10461617) polymorphism and T2DM in a sample of Iranian subjects. The homozygous AA genotype had higher T2DM risk compared with heterozygous AG genotype [324]. Transforming growth factor-b (TGF-b) is a member of growth factors family, which have important regulatory impacts on many physiological processes [325]. TGF-b/Smad3 signaling is a pivotal regulator of insulin expression that can be deregulated in diabetes [326]. It has been observed that TGF-b1 (T869C) C and T alleles increased and reduced T2D susceptibility among a sample of Egyptian patients, respectively [327].

### Structural proteins

The function and structure of many cell types are associated with the extracellular matrix that is involved in cell adhesion, cellular differentiation, proliferation, and migration [328]. Noticeable modifications in the structure and synthesis of the extracellular matrix have been reported in diabetes mellitus. Hyperglycemia and insulin resistance were reported to be correlated with collagen IV levels [329]. Zinc-dependent endopeptidases are called matrix metalloproteinases (MMP) that affect matrix and non-matrix proteins [330]. A study was done in an Iranian population to examine the probable correlation between COL4A3 (rs55703767, G/T) and MMP-9 (rs17576, A/G) polymorphisms and T2D. T allele of COL4A3 (G/T) had a protective role, whereas A allele of MMP-9 (A/G) appeared to be a risk factor of T2D [331]. Serine protease inhibitor B1 (SerpinB1) acts as a neutrophil elastase inhibitor, which is correlated with improved insulin sensitivity [332, 333]. It also inhibits cell migration by MMP-2 down-regulation [334]. Diabetic cases with rs15286 AA genotype had higher HOMA2-β levels and lower FPG and HbA1C levels, compared with other genotypes. There was also a significant correlation between AA genotype and good glycemic control among Egyptian patients. Moreover, there was a direct correlation between G allele and prediction of poor glycemic control [335]. Calpains are cysteine proteases involved in cell proliferation, signal transduction, apoptosis, membrane fusion, and platelet activation [336, 337]. Calpain 10 (CAPN10) regulates the reorganization of actin which is vital for insulin-stimulated translocation of GLUT4 to the plasma membrane of adipocytes [338]. There was a significant correlation between allele 2 (C) of CAPN10 (SNP-44) and increased risk of T2DM in a sample of Palestinian cases [339]. There was also a correlation between SNP-44 polymorphism and T2DM in a sample of Turkish cases in which T allele had lower frequency among patients compared with control group [340].

Engulfment And Cell Motility 1 (ELMO1) is involved in cell movement and phagocytosis [341]. Correlation between the re1345365, rs741301 variants, and DN were assessed in an Iranian subpopulation. There was an association between allelic and genotypic frequencies of the rs741301 variant and DN. G alleles and GG genotype carriers had higher DN susceptibility. The rs1345365A/rs741301G was considered as a risk haplotype for DN progression in T2DM patients [342]. There was also a significant correlation between ELMO1 gene (rs741301) polymorphism and DN in a sample of Egyptian subjects. DN patients with GG genotype and G allele were more than twice as likely to develop DN. The ELMO1 (rs741301) polymorphism increased the DN susceptibility among T2D patients [343]. SNARE protein family including VAMP2 and SNAP25 are structural proteins involved in insulin secretion through the vesicle fusion. It has been shown that SNAP25 polymorphisms were associated with the concentration of HbA1c, fasting glycemia, and insulinemia in T2DM patients. There were also significant increased levels of HbA1c and fasting glucose among diabetic patients who were carriers of the rs363050 (AG/GG) compared with (AA) genotype. Insulin levels were significantly higher in carriers of the (AA) genotype compared with (AG/GG) [344].

ERBB receptor feedback inhibitor 1 (ERRFI1) is an adapter protein involved in regulation of tyrosine kinase receptors [345–347]. Over its antagonistic role in the EGFR signaling pathway, ERRFI1 appears to decrease the mass of beta cells [348, 349]. A study was done in a group of Iranian diabetic cases to examine the correlation between + 808 (T/G) polymorphism and DN. There was a remarkable correlation between + 808 T/G variant and diabetes. T allele of this polymorphism had a protective role against diabetes [350].

### Renin–angiotensin system

The renin–angiotensin system (RAS) includes a series of cellular processes that lead to the generation of angiotensin II. The activation of this system has a pivotal role in CAD and hypertension [351, 352]. The correlation between AT1R A11166C polymorphism and DM was assessed in a group of Iranian subjects with CAD. There was a significant higher frequency of polymorphic genotypes (AC and CC) and the 1166 C allele in the diabetic group compared with non-diabetic cases [353]. The angiotensin-converting enzyme (ACE), which is a vital part of the RAS, is involved in the homeostasis of renal electrolytes and regulation of systemic blood pressure [354, 355]. There was significant correlation between DD genotype and D allele of ACE and increased T2DM progression among Egyptian and Saudi Arabian cases [356, 357]. There were also high frequencies of D allele and DD genotypes of ACE I/D polymorphism among Kuwaiti T2DM patients [358].

## Conclusions

Diabetes is a chronic disorder that often lacks any significant clinical symptoms in the early stages. Therefore, late diagnosis can be associated with tissue damages in various organs such as kidney and cardiovascular systems that leads in diabetic complications. SNPs are pivotal factors involved in diabetic susceptibility that can be used for the early detection and better disease management. Given the high prevalence of diabetes in Middle East, in the present review we assessed the role of SNPs in diabetes susceptibility and prevalence in this region. It has been shown that the diabetes-related SNPs were mainly observed in genes which were associated with immune system, nuclear receptors, and insulin signaling pathway. Since, various SNPs have been reported in different Middle East countries, it is difficult to introduce an efficient general SNP-based diagnostic panel marker in this region. However, based on the number of studied patients in this region, it seems that a general panel of NOS, TCF7L2, VDR, and PON1 polymorphisms can be used as diagnostic panel markers to identify the susceptible cases to diabetes in Middle East population. Moreover, we can also introduce TNF-α (-308G/A), NPC1(rs1805081 and rs1788799), MPO (-463G/A), TCF7L2 (rs4506565 and rs12255372), KCNJ11 (rs5219), IGF2BP2 (rs4402960 and rs1470579), VDR (rs10735810, rs731236, rs7975232, and rs1544410), TCF7L2 (rs7903146), eNOS (T786C and G894T), and ACE (C677T and I/D) polymorphisms as the candidates for the screening of the diabetes susceptibility among Iranian, Saudi Arabia, Turkish, Emirati, Syrian, Lebanese, Kuwaiti, Palestinian, Jordanian, and Bahraini populations, respectively. Regarding the high racial diversity in the Middle East countries, the present review can be considered as a suitable model to investigate the role of SNPs in other races and countries to pave the way of introducing a global SNP-based diagnostic panel marker for diabetes.

## Data Availability

The datasets used and/or analyzed during the current study are available from the corresponding author on reasonable request.

## Abbreviations

SNPs: Single-nucleotide polymorphisms
GDM: Gestational diabetes mellitus
T1D: Type 1 diabetes
T2D: Type 2 diabetes mellitus
ADIPOQ: Adiponectin
MIF: Macrophage migration inhibitory factor
HLA-G: Human leukocyte antigen-G
VDR: Vitamin D receptor
ER: Estrogen receptor
SHBG: Sex hormone-binding globulin
PPAR-γ: Peroxisome proliferator-activated receptor-γ
GST: Glutathione S-transferases
ROS: Reactive oxygen species
NODM: New-onset diabetes mellitus
FFAs: Free fatty acids
SOD: Superoxide dismutase
mEPHX: Microsomal epoxide hydrolase
HSPs: Heat-shock proteins
NAT1: N-acetyltransferase 1
PON1: Paraoxonase 1
CAT: Catalase
GPx: Glutathione peroxidase
MPO: Myeloperoxidase
IRS1: Insulin receptor substrate protein 1
SH2B1: Src-homology-2 B adaptor protein 1
AHSG: Alfa2-Heremans-Schmid glycoprotein
SEPP1: Selenoprotein P
OCTs: Organic cation transporters
ER: Endoplasmic reticulum
UCP2: Uncoupling protein 2
GIT: Gastrointestinal tract
GIP: Glucose‐dependent insulinotropic polypeptide
GLP-1: Glucagon-like peptide-1
miRNAs: MicroRNAs
ANGPTLs: Angiopoietin-like proteins
CETP: Cholesteryl ester transfer protein
FADS1: Fatty acid desaturase 1
PLIN: Perilipin
PKA: Protein kinase A
HSL: Hormone-sensitive lipase
DN: Diabetic nephropathy
APOA5: Apolipoprotein A5
NO: Nitric oxide
NOSs: Nitric oxide synthases
ESRD: End-stage renal disease
TCF7L2: Transcription factor 7-like 2
HHEX: Hematopoietically expressed homeobox
TGF-b: Transforming growth factor-b
MMP: Matrix metalloproteinases
SerpinB1: Serine protease inhibitor B1
CAPN10: Calpain 10
ELMO1: Engulfment and Cell Motility 1
ERRFI1: ERBB receptor feedback inhibitor 1
RAS: Renin–angiotensin system
CAD: Coronary artery disease
ACE: Angiotensin-converting enzyme
